# The Route of the Malignant Plasma Cell in Its Survival Niche: Exploring “Multiple Myelomas”

**DOI:** 10.3390/cancers14133271

**Published:** 2022-07-04

**Authors:** Antonio Giovanni Solimando, Matteo Claudio Da Vià, Niccolò Bolli, Torsten Steinbrunn

**Affiliations:** 1 Department of Biomedical Sciences and Human Oncology, Section of Internal Medicine ‘G. Baccelli’, University of Bari Medical School, 70124 Bari, Italy; 2 Department of Medicine II, University Hospital of Würzburg, 97080 Würzburg, Germany; 3 Hematology Unit, Fondazione IRCCS Ca’ Granda Ospedale Maggiore Policlinico, 20122 Milan, Italy; matteo.davia@unimi.it (M.C.D.V.); niccolo.bolli@unimi.it (N.B.); 4 Department of Oncology and Hemato-Oncology, University of Milan, 20122 Milan, Italy; 5 Department of Medical Oncology, Dana-Farber Cancer Institute, Harvard Medical School, Boston, MA 02215, USA

**Keywords:** multiple myeloma, cell of origin, cancer stem cells, bone marrow homing, adhesion molecule, bone marrow immune-microenvironment

## Abstract

**Simple Summary:**

Multiple Myeloma is a hematological neoplasia originating from malignant plasma cells in the bone marrow. Despite improved therapies, this cancer is still considered incurable due to the occurrence of relapse and the development of drug resistance over time. Apart from cell-intrinsic oncogenic features such as genetic alterations and clonal evolution that promote tumor growth and survival, increasing evidence demonstrates that the bone marrow microenvironment plays a pivotal role in disease relapse and immune evasion by providing protected bone marrow niches in which dormant myeloma cells are able to reside and survive. This review summarizes the ways of the bone marrow micromilieu to nurture and interact with the malignant plasma cells, and provides insights into the vicious cycles arising from the interplay between myeloma cells and their surrounding tumoral stroma. Knowledge about these mechanisms and how to disrupt them may provide novel approaches to targeting and tackling multiple myeloma.

**Abstract:**

Growing evidence points to multiple myeloma (MM) and its stromal microenvironment using several mechanisms to subvert effective immune and anti-tumor responses. Recent advances have uncovered the tumor-stromal cell influence in regulating the immune-microenvironment and have envisioned targeting these suppressive pathways to improve therapeutic outcomes. Nevertheless, some subgroups of patients include those with particularly unfavorable prognoses. Biological stratification can be used to categorize patient-, disease- or therapy-related factors, or alternatively, these biological determinants can be included in a dynamic model that customizes a given treatment to a specific patient. Genetic heterogeneity and current knowledge enforce a systematic and comprehensive bench-to-bedside approach. Given the increasing role of cancer stem cells (CSCs) in better characterizing the pathogenesis of solid and hematological malignancies, disease relapse, and drug resistance, identifying and describing CSCs is of paramount importance in the management of MM. Even though the function of CSCs is well-known in other cancer types, their role in MM remains elusive. With this review, we aim to provide an update on MM homing and resilience in the bone marrow micro milieu. These data are particularly interesting for clinicians facing unmet medical needs while designing novel treatment approaches for MM.

## 1. Introduction

Multiple myeloma (MM) is a malignant proliferation of clonal plasma cells (PCs) that expand in the bone marrow (BM), with ensuing induction of focal skeletal lesions and osteoporosis. Clinical presentation includes myeloma bone disease, anemia, renal insufficiency, hypercalcemia [1,2], higher infection rates [3,4] and secondary life-threatening complications [5,6,7,8]. MM remains an incurable disease, despite conventional therapies [9,10] and considerable improvements in novel therapeutic approaches [11]. The MM cell within the bone niche has direct and indirect contact with skeletal cells modulating bone resorption and regeneration [12]. However, single cells may also persist and inhabit protected BM niches, which could serve as a cause for residual disease and relapse [13,14]. Physical interaction between skeletal precursors and MM cells creates niches and influences bone cells (mesenchymal stem cells (MSCs) and osteoblasts (OBs)). Single-cell harvesting and OMICs analyses have uncovered that the mutual interaction of tumor and bone is critical in the niche and tumor microenvironment (TME) [15], where several vicious cycles nurse the MM cells [16,17,18,19]. Biological dissection of the BM niche of normal vs. malignant PCs revealed that the niche constitution and interaction factors are related to different clinical portraits and are connected to the malignant PCs by molecular profiling—this highlights novel biological taxonomies of multiple myelomas [20,21].

Single-cell analyses of this molecular crosstalk have revealed new targets [22,23,24], which could mobilize and eradicate niche-protected dormant and putative cancer myeloma stem cells (CSCs). The primary application of such discoveries would be to cure residual disease and reactivate bone regeneration by hitting both the MM and its bystander counterparts [25,26,27]. Indeed, MM progresses through constant crosstalk with the surrounding milieu, and recent reports also implicate aberrant angiogenesis and immunosuppression, which simultaneously support MM progression and related CSC nursing [28,29]. As such, in vitro [30], in vivo [31], in the embryo [32,33], ex vivo [34] and in silico [35,36] strategies that combine anti-angiogenic therapy [37,38,39] with immunotherapy [40,41] reportedly tip the balance of the TME, thereby amplifying treatment response. Here, we review unexplored biological characteristics that act in the dissemination of myeloma, with a particular focus on the putative cell of origin of MM.

## 2. Cell of Origin, Homing and Soluble Cytokines in Multiple Myeloma

Even though substantial work on the development of MM and the mechanism of drug resistance has elucidated many aspects of disease biology, MM remains chronic and incurable neoplasia, and the lack of therapeutic strategies to reach and eliminate the “cell of origin” remains an urgent unmet clinical need. According to the hypotheses formulated on the origin and evolution of MM, the myelomatous precursors, which putatively are pre-B cells with clonogenic properties (CD19+, CD38+ and CD56+), circulate throughout the bone marrow and penetrate the vascular endothelium by anchoring to specific adhesion molecules that belong to the integrin family (ICAM-1, ICAM-2, VCAM and JAMs) [42,43,44].

Plasma cell malignancies are closely related to the state of differentiation within which the malignant evolution occurs [45,46]. The cell differentiation process requires participation by RAG1 and RAG2—these proteins physically recombine different segments, including the Variable (V), Diversity (D) and Joining (J) segments, of the immunoglobulin-encoding genes. RAG 1 and 2 proteins play a pivotal role in recognizing and excising the recombination signal sequences that comprise conserved heptamer and nonamer sequences separated by a spacer. The recombined IgH gene is transcribed, translated and expressed on the cell surface with a light chain surrogate [47,48]. Cell membrane heavy and light chains, as well as the B-cell receptor (BCR), distinguish the immature B cells stage, after which B cells migrate from the bone marrow into the periphery and secondary lymphoid tissues and undergo maturation.

**Figure 1:**
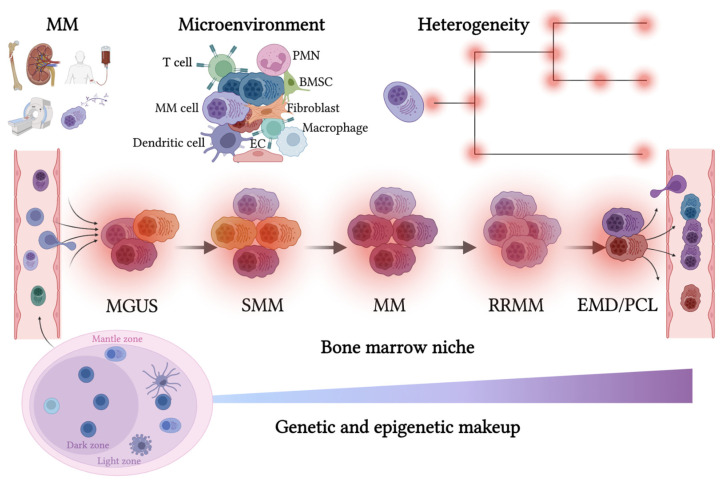
**The route of the malignant plasma cell in its survival niche.** Multiple myeloma disease trajectory is schematized. Upper panel, left, clinical C.R.A.B. (hypercalcemia, renal failure, anemia, and bone disease; additional diagnostic criteria S.Li.M.—sixty percent clonal bone marrow infiltration, ratio light chains involved/uninvolved greater than 100, more than one focal lesion detected with MRI) results from cell-extrinsic and MM-intrinsic factors, driving disease progression. Left side, bottom: germinal center with B cells, plasma cells, and follicular dendritic cells; top: immune-microenvironment infiltration (left) and scheme of multiple alterations building heterogeneous genomic architecture (right). MM: multiple myeloma; PMN: neutrophil granulocytes; BMSC: bone marrow stromal cell; EC: endothelial cell; MGUS: monoclonal gammopathy of undetermined significance; SMM: smoldering multiple myeloma; EMD: extramedullary disease; PCL: plasma cell leukemia. Created with BioRender.com.

B cell activation can occur by antigen stimulation via T cell-dependent (TD) cytokines, triggering complex B-cell signaling that results in the selection of B cells with higher-affinity B-cell receptors and longer-lasting immunity [49,50]. This process is critical for the onset of MM due to a strict collaboration between T and B cells [51,52]. While triggering a cascade of events, including the expression of the CD40 ligand, B cell signaling induction, and the release of IL-4, IL-21 and IL-6 [46], T cell cytokines help to trigger the B cell activation while inducing rapid proliferation by constituting a lymphoid structure known as the “germinal center” [52] (Figure 1). Within the germinal center reaction, B cells continuously cycle through rounds of division and selection for high-affinity antibodies, which are made through somatic hypermutation (SHM) and class-switch recombination (CSR). The activation-induced cytidine deaminase (AID) orchestrates both phenomena [46].

AID deaminates cytosines on single-stranded DNA, which leads to mutations in both the immunoglobulin heavy and light chains. SHM of the heavy and light chains can enhance antibody–antigen affinity through the mutation of the complementarity determining region. This leads to increases T cell stimulation and selection of B cell clones with high-affinity antibodies to a given antigen and results in more efficient antigen uptake and presentation [53]. CSR occurs depending on IgH somatic recombination within the constant region µ, and its splice isoform δ, with one of the alternative constant regions α1,2, ε or γ1–4, via an AID-dependent switch region recombination [54]. Substantial evidence now points to MM-initiating alterations arising from errors in CSR.

Bone marrow and the fetal liver, on the other hand, are the first niches in which the primitive hematopoietic stem cells are nursed, after which these stem cells differentiate into multipotent cells, common lymphoid precursors and ultimately mature B cells through pre-pro-B, pro-B, pre-B, immature B and transitional B cells [55]. A reciprocal interaction between the pre-myelomatous and stromal cells takes place at these two sites and feeds into a vicious cycle that involves increased autocrine and paracrine production of IL-6 and other cytokines, which are pivotal for MM cell maturation and subsequent abnormal proliferation [55,56].

Exogenous IL-6 added to human MM cell lines is essential for developing myelomatous cells in vitro [57]. As mentioned above, this activates the protein gp130, leading to phosphorylation of downstream kinases. Of note, IL-6 tips the balance between hyperdiploid (HMM) and non-hyperdiploid MMs while delineating a gene expression fingerprint within the HMM [58]. IL-6 is also correlated with oncogenic signaling, which fuels the transition from a long-lived plasma cell to a full-blown MM cell [59,60,61]. Ciliary neurotrophic factor (CNTF), leukemia inhibiting factor (LIF), IL-11 and oncostatin M (OSM) boost IL-6 activity [62,63] and are the main downstream effectors, pointing to a potential theragnostic window, which could be exploited in treating the early phases of MM development [63] and supporting IL-6-mediated support for MM growth and survival [64]. Indeed, glycoprotein 130 (gp130) is crucial for IL-6 signaling [63]. Of note, in long-term and high cell density cultures, MM cells become emancipated from the surrounding niche and can support autocrine IL-6 production. In conclusion, activation of gp130 constitutes a fundamental event in the survival and growth of MM cells to such an extent that dimerization can be induced by various cytokines such as IL-6 and IL6-related cytokines [63].

Interferon (IFN)-alpha, nuclear factor (NF)-kappa-B and granulocyte-macrophage colony-stimulating factor (GMCSF) guarantee the survival of myelomatous cells and G-CSF, IL-1, IL-3 and IL-5 also play a key role in plasma cell growth and differentiation [65]. These soluble factors fuel MM cell survival by modulating key processes such as cell death and autophagy flux [66] and can be therapeutically targeted directly [67] as well as indirectly [68,69]. Other soluble factors participating in the interplay between the MM cells and the cellular TME include insulin-like growth factor (IGF)-1, vascular endothelial growth factor (VEGF), tumor necrosis factor (TNF)-alpha, B cell-activating factor, a member of the TNF family (BAFF), fibroblast growth factor (FGF) and stromal cell-derived factor (SDF)-1-alpha and also contribute to the development of drug resistance [70]. Gaining a deeper insight into the interaction between MM cells, immune cells and bone cells is especially important because the immune and non-immune cells hold great potential for eliminating malignant cells [71]. However, the regulation and therapeutic activation of anti-proliferative and homing responses in the bone marrow are understudied, and novel strategies aimed at halting myeloma and its associated bone disease are still urgently needed.

Apart from the MM cell-intrinsic factors mentioned above that promote MM cell survival and increase resistance, efforts are also underway to characterize extrinsic influences that can be exploited for therapeutic interventions. The bone marrow microenvironment of MM patients displays altered phenotypes, and comparisons of single-cell transcriptomics of MM cells and their stromal microenvironment show inflammatory stromal signatures that are specific for MM [72]. The medullary microenvironment comprises various extracellular matrix proteins, including fibronectin, collagen, laminin and osteopontin along with cellular components that include stem cells, progenitor cells and hematopoietic precursors, as well as stromal cells such as immune and endothelial cells, fibroblasts, osteoclasts and osteoblasts [13,21,27,73]. The physical interaction between MM cells, extracellular matrix and accessory cells plays a crucial role in the pathogenesis of MM and can be leveraged for developing therapeutics [24,74]. In particular, the direct interaction between MM cells and accessory cells, and the subsequent production of cytokines, activates signaling pathways that mediate the growth, survival and migration of myelomatous cells and also support osteoclastogenesis and angiogenesis [75,76,77]. As a result, the BM microenvironment becomes more prone to accepting and supporting myelomatous precursors, which lose the ability to divide and migrate. On the other hand, the myelomatous mass grows and expands due to the continuous recruitment of plasma cell precursors. The MM microenvironment in the different disease stages [21] shapes the disease’s immune phenotype, characterized by divergent expression in NK cells, cytotoxic T cells and monocytic cells across the MM trajectory. In this context, dormant MM cells, i.e., non-proliferating MM cells that are arrested in the G0–G1 phase of the cell cycle, directly interact with the immunological niche via PD-L1 and MHC II molecules, regulate the neoplastic load, modulate the disseminative potential and even anticipate clinically measurable disease. Specifically, genomics uncovered that MGUS/SMM clones harbor chromosomal alterations that define MM translocations involving IgH or hyperdiploidy. However, not all patients with MGUS/SMM who harbor a similar genetic architecture eventually progress to MM. Indeed, while priming a corrupted microenvironment, the complex ecosystem can determine a reduced anti-tumor response, induction of angiogenesis, resistance to therapy and disease progression [21,75]. MM cells are nursed by an immunosuppressed milieu, stimulated by the neoplastic PCs resulting in an expansion of regulatory T cells and an increase in myeloid-derived suppressor cells while fueling a dysfunction of tumor-associated macrophages and NK cells [13,21].

In the MGUS stage, a significant enrichment is detected in NK, T and CD16+ cells in the diseased BM, and a relative decrease in plasmacytoid dendritic cells, immature neutrophils, CD14+ monocytes and other bystander cells, which together shape a permissive environment [19,27,78]. Therefore, the MM quiescent stage can anticipate the full-blown disease, and the dissemination within and outside the bone marrow is thus an inefficient process in which some MM cells overcome the boundary of the detectable symptomatic systemic threshold, while other malignant foci persist and remain dormant for a protracted length of time [79].

The concept of dormant cancer cells that refers to non-proliferating MM cells arrested in the cell cycle’s G0–G1 phase is vital for highlighting the temporary mitotic arrest. In this context, cancer cell dormancy could result from the seemingly spontaneous reactivation of therapy-resistant, dormant cancer cells. However, the life cycle of the dormant cancer cell leads to a niche occupancy that requires disseminated cancer cells to find a supportive milieu. Next, the predilection to home towards a given site, based on the tissue of origin and histological subtype of the primary tumor, points towards the cells’ journey, which consists of interactions and engaging with the niche [79]. Crucial factors at this interface, such as oxygen, regulate cell proliferation and thereby promote drug resistance, and compelling evidence in MM implicates the niche in inducing dormancy [14]. Cellular reprogramming and environmental adaptation prompted further investigations regarding the primary factors that tip the balance between dormant cancer cells’ pre-programmed characteristics and dormancy induction by the niche. Although conflicting data exist that elucidate this vicious cycle flux, the establishment of long-term dormancy guides a complex cellular reprogramming aimed at the evasion of immunosurveillance by dormant MM cells [80].

The subsequent stage leads to the reactivation of dormant MM cells in the niche, depending on hypoxia, metabolic rate and additional extrinsic factors. Within the bone microenvironment, resorption of osteoclastic bone and osteoclast-mediated remodeling disrupt the cellular interactions that hold cancer cells in a dormant state, releasing the cells from niche-dependent control [79]. In this complex scenario, asymptomatic clonal entities usually precede cancer, suggesting an advantage of subclones that accumulate genomic driver events over time and allowing their expansion and progression. Thus, it is vital to develop suitable strategies to sketch the disease trajectory better, hijack MM evolution and prevent and diagnose early premalignant clonal initiation and progression [81].

## 3. Myeloma Stemness, Genomic Evolution and Clinical Implications

The emerging role of adhesion molecules in driving the behavior of aggressive diseases has prompted a deeper characterization of biological and translational implications of interactions between PCs and the TME. Adhesion of malignant myeloma cells to the BM stromal cells, plus the extracellular matrix, contributes to drug resistance by activating anti-apoptotic and pro-survival pathways [70]. Both JAM-A and CD44 are involved in the maintenance of cancer stemness [82,83,84,85,86,87]. Molecular events lead to the generation of the PCs that initiate MMs. Targeting JAM-A and CD44 not only threatens the self-renewal of CSCs [82,83,84] but also overcomes drug resistance [88]. MM stem cell-like phenotype [89], transformation into non-secretory disease, or development into light chain disease are associated with more aggressive clinical features, frequent DR and worse clinical outcomes [90]. Somatic immunoglobulin gene hypermutation without intraclonal variation is a hallmark of MM PCs [91]. Chromosomal translocations that involve the IgH locus (14q32) and hyperdiploidy constitute ancestral genetic lesions [11,12], while secondary alterations occur during disease progression. Chromosomal translocation t(11;14) reportedly is found in MM patients [92,93] and may be a marker of the initially immortalized clone, suggesting that MM arises from a post-germinal center B cell compartment. Moreover, CD19+ B cells isolated from MM patients can form a new tumor in xenograft models, suggesting the existence of cells’ self-renewal [43]. Highly clonogenic MM cells lack the CD138 expression that is typical of PCs but express CD20, druggable targets [94,95] and surface immunoglobulins [43,96].

Collectively, the above findings argue for a deep-level diagnostic work-up of patient samples, using next-generation sequencing to identify mutational profiles and multiparametric flow cytometry for deep immunophenotyping. A detailed molecular definition—down to the single-cell level—will enable a better understanding of the clonal and sub-clonal evolution of MM and a better stratification of individual MM cases [93].

MM PCs have been hypothesized to originate in BM hematopoietic stem cells (HSCs). Stem cells can self-renew, and they exhibit asymmetric division [89]. Simultaneously, mesenchymal stem cells (MSCs) have been found close to HSCs, and support cell growth. MSCs exhibit a large variety of surface markers, including CD29, CD44, CD49a–f, CD51, CD73, CD105, CD106 and CD166, but not some typical hematopoietic lineage markers such as CD11b, CD14 and CD45 [96,97,98,99,100]. On the other hand, BM-MSCs from MM patients differ from the healthy donor MSCs, in that they have low expression of fibronectin and VCAM-1. MSCs potentially drive malignant PCs’ BM localization and retention [97,98,99,100].

Despite considerable evidence on well-characterized CSCs in solid and hematological neoplasms, information about potential CSCs in MM is scarce [100]. Because CD19- and CD27-positive circulating cells display clonotypic properties, the post-germinal center compartment is thought to be the source of MM CSCs [100]. This memory B cell-like population (CD19+CD27+CD138 neg) has sufficient clonogenic capacity to self-renew and sustain MM expansion [97,100] (Table 1). Therapeutic consequences appear to parallel this progenitor architectural view of MM cells by linking drug resistance to the presence of tumoral B cells and pre-plasmablasts that have low sensitivity to conventional and novel anti-MM agents [101]. The MM cell taxonomy contributes to proteasome inhibitor resistance. Indeed, tumor B cells and pre-plasmablast that are negative for the spliced isoform of Xbp1s, a transcription factor required for PC differentiation, have a survival advantage when they are subjected to proteasome inhibition. At the same time, the maturation arrest of cells at the stage before the plasmablasts of MM cells enables progressive disease after treatment with a proteasome inhibitor. Xbp1s can feed the decommitment from PC maturation and immunoglobulin production and can diminish endoplasmic reticulum and cytotoxic susceptibility [101].

Multiple molecular events contribute to malignant PC transformation during disease pathogenesis [102] through a branching process in which the subclonal composition of MM changes over time [103] and is characterized by a hybrid genomic architecture [104]. Even though all patients display intratumor heterogeneity, RAS mutations may represent the true driver mutations in MM [105]. The relevance of RAS as one of the most frequent secondary lesions has also been confirmed in MM [92].

Chapman et al. first described the genomic landscape for MM based on data derived from a WES analysis of 24 MM patients [102]: they highlighted a specific genomic signature, which was later confirmed by two extensive studies led by Bolli and Lohr [106,107]. The co-mutational status of driver genes in MM that involve KRAS, NRAS and BRAF has led to novel targeted therapies. A deeper understanding of the clonal heterogeneity, especially at the sub-clonal level, could yield a biological rationale for combining multi-targeted therapy to trigger a more robust response [107]. KRAS/NRAS mutations constitute a milestone in disease progression because these mutated oncogenes can drive clonal expansion and competition during the process of drug-selective pressure [108,109]. Melchor et al. have correlated oncogenic RAS to mutations involving IRF4, a transcription factor critical for controlling B-cell proliferation and differentiation [92], and the RAS pathway has been linked to disease pathogenesis, underscoring the role of adhesion molecules in the progression of MM [110,111,112]. Adhesion molecules [87,113] are involved in CSC maintenance and thus may be involved in sustaining the progression of RAS-related and RAS-independent diseases during clonal evolution [114,115,116].

Despite efforts to clarify the multistep molecular disease evolution, the peculiar sub-clonal genomic heterogeneity that characterizes MM [106] must be acknowledged. This complexity has been dissected in a cohort of 67 MM patients, in whom four patterns of clonal evolution were identified: (i) the different clonal and sub-clonal populations were equally affected by the treatment and maintained the ability to repopulate the BM after the selective pressure therapy; (ii) the patients’ clonal and sub-clonal heterogeneity was maintained, and only the proportion of the different populations changed; (iii) new sub-clones appeared, which were undetected before the treatment; (iv) the “branching evolution” was seen, where, through therapeutic interventions, specific clones were selected and eliminated, giving rise to other previously undetected clones which grew and proliferated, causing the progression of the disease [106]. These findings are summarized in Figure 1.

An urgent unmet clinical need is the translation of this deep molecular characterization. A “static progression and spontaneous evolution model” [117] for further disease progression at the genomic dissection level has been identified. Progressive accumulation of disease burdens plays a central role in maintaining a sub-clonal architecture, and conversely, the various changes in MM composition form the backbone of spontaneous evolution [117].

These findings are supported by current reports that MM is most likely correlated with a truncal event within the germinal center during the fourth decade of life [81]. They also have some translational consequences. Because Wnt-signaling is correlated with cancer cell stemness [118] and MM disease progression [119], several therapeutic approaches have been employed in MM [120], as well as in several other hematological [121] and solid malignancies [122,123]. The Wnt pathway regulates cell migration and differentiation. In MM, this pathway is activated by the interplay between PCs and the BM microenvironment (Figure 1). Activation of Wnt can influence several intracellular pathways, including the canonical Wnt/β-catenin and the non-canonical Wnt/Ca2+ pathways. Genetic modifications of Wnt signaling induce down-regulation of pathway activity and support the proliferation of MM cells. The use of pharmacological small molecule inhibitors to target the Wnt pathway can disrupt MM cell maintenance, both in vitro and in vivo, thus opening a promising therapeutic window [124].

The BCL2 pathway is one of the most promising target pathways in hematological malignancies, particularly in the peculiar t(11;14) setting of MM. Kumar et al. have uncovered venetoclax to be therapeutically active as a single agent in t(11;14) RRMM, with a 21% ORR. Stratifying for patients by using t(11;14) as class boundary, ORR even reached 40% [125]. By inhibiting the physiological process of eliminating damaged and stressed cells, venetoclax restores the programmed cell death equilibrium. Therefore, assessing BCL2 expression while stratifying subjects depending on the presence of t(11;14) holds the potential of corroborating the response prediction to venetoclax [125]. Additionally, venetoclax enhances the activity of bortezomib in MM cell lines and NCI H929 xenograft models, pointing toward an additive effect exerted by BCL2 inhibition and MCL-1 blocking by binding the BCL2-family member NOXA in a bortezomib-dependent fashion [126,127].

As in other cancer entities, the availability of CRISPR-Cas9-based genome editing technologies has opened revolutionary ways of understanding MM biology and translating these findings into clinical studies. Using comprehensive CRISPR-Cas9 knockout screens in vitro, Bohl et al. characterized relapse-specific mutations associated with resistance of MM cells against clinically applied drugs [128]. These screens could also identify previously unknown vulnerabilities towards substances that constitute a therapeutic option for distinct MM patients. A recent clinical phase 1 trial included two patients with advanced therapy-refractory MM, in whom the utility of CRISPR-Cas9-mediated silencing of the immune checkpoint PD-1 was tested in patient-derived T cells, together with simultaneously disrupting their T cell receptor function [129]. The modified T cells persisted in patients for at least nine months and were well tolerated. An ongoing phase 1 clinical trial is being carried out in relapsed/refractory MM patients, using allogeneic CRISPR-Cas9-engineered T cells targeting BCMA, and may reveal future ways to an “off-the-shelf” product (NCT04244656).

Growing evidence documents that “multiple myelomas” and their stromal microenvironment have a multitude of mechanisms to subvert effective immune and anti-tumor responses (Figure 1) [95,130,131]. Understanding how tumor and stromal cells (inter-)act to modulate and regulate the immune-microenvironment should facilitate the development of therapeutic interventions that target these suppressive pathways and may be harnessed to improve therapeutic outcomes in the heterogeneity of MM disease phenotypes [108,132]. MM grows and evolves through persistent crosstalk with the surrounding niche, while distinct gatekeepers and immune-tolerogenic environments often emerge simultaneously in response to this crosstalk. Accordingly, combining anti-tumor therapy and immunotherapy could be a promising and effective strategy to slope the equilibrium of the MM ecosystem, and improve patient outcomes, as already obtained in other malignancies [133,134,135,136,137,138]: this approach will enable the development of personalized therapy for “multiple myelomas” [139,140].

## 4. Conclusions

Despite our knowledge about the vicious cycles within MM, mediated by malignant plasma cells, osteoblasts and osteoclasts, it is vital to gain further insights into the cellular and molecular mechanisms of the interaction between MM cells and bone cells within the bone microenvironment as well as the process of cell colonization in the bone. Deep-diving into epigenetics and subsequent characterization of miRNAs and non-coding transcripts have unraveled novel mechanisms of gene expression regulation, often involving the multiple myeloma microenvironment, and warrant further translational application.

## Figures and Tables

**Table 1:** Immunophenotype dissecting B-cell differentiation and myeloma evolution.

Cell Type	Cell Marker
Premalignant plasma cell	CD38+; CD19−; CD27+/−; CD20−; CD56+/−
Malignant plasma cell	CD38+; CD19−; CD27+/−; CD20−; CD56+/−
Clonotypic centroblast/centrocytes	CD38+; CD20+; CXCR4+/−
Clonotypic memory B-cell	CD38+; CD20+; CD27+; mIgG/A
Clonotypic plasmablast	CD38+; CD20+; CD27+
Human myeloma cell lines	CD38+; CD19−; CD27+/−; CD20-; CD56+/−
